# Implementation of a top five list to identify medical overuse in general practice according to patients’ viewpoint in 2019 in France

**DOI:** 10.1186/s12875-021-01475-z

**Published:** 2021-06-26

**Authors:** Agnès Hazard, Marion Debin, Corentin Hervé, Caroline Guerrisi, Camille Bonnet, Mathilde François

**Affiliations:** 1 Department of Family Medicine, Faculty of Health Sciences Simone Veil, University Versailles-Saint-Quentin-en-Yvelines, Villejuif, Paris, France; 2 Sorbonne Université, INSERM, Institut Pierre Louis D’Epidémiologie Et de Santé Publique, IPLESP F-75012 Paris, France; 3 Centre for Research in Epidemiology and Population Health, French National Institute of Health and Medical Research (INSERM U, University Versailles Saint-Quentin-en-Yvelines, University Paris-Sud, 1018), Villejuif, France

**Keywords:** Medical Overuse, General practice, France, Patient

## Abstract

**Background:**

There is a current trend to reassess the adequacy of care. Establishing top five lists by involving patients is one way to address medical overuse. The objective of this study was to establish a patients’ top five list in general practice in France. The secondary objective was to identify selection criteria.

**Method:**

Patients from the web-based cohort GrippeNet.fr were invited to establish their top five list from 15 care procedures previously selected by general practitioners on the basis of medical overuse. The care procedures were presented on a web-interface with guides written with the help of a patient association. A questionnaire was used to explore factors that may have influenced the choices of the participants.

**Results:**

In total, 691 patients established the following top five list: 1/ Prescription of antibiotics for acute bronchitis, nasopharyngitis, otitis media with effusion, or uncomplicated influenza; 2/ Prescription of benzodiazepine and benzodiazepine-like agents for insomnia, generalised anxiety and all indications for older patients; 3/ Prescription of a homeopathic treatment (Influenzinum) for flu prevention; 4/ Prescription of antitussive or expectorant agents for acute cough or acute bronchitis care; 5/ Prescription of statins for the primary prevention of cardio-vascular risk in older patients. More than 70% of participants gave importance to the recommendations, effectiveness, and tolerance of the care procedures, whereas only half considered the cost.

**Conclusion:**

This study is the first to establish a patient’s top-five list in general practice. This list provides direction for deciding the main targets in limiting medical overuse.

**Supplementary Information:**

The online version contains supplementary material available at 10.1186/s12875-021-01475-z.

## Introduction

There is currently a trend to limit medical overuse and reassess the adequacy of care to protect the health of patients and make better use of financial resources [1, 2]. The most consensual definition of medical overuse is "a healthcare service [that] is provided under circumstances in which its potential for harm exceeds the possible benefit" [3]. This phenomenon is multifactorial and involves both general practitioners (GPs), patients, and healthcare systems [4]. According to doctors, the main cause of medical overuse comes from patient requests [5], with multiple consequences, the main one being the deleterious effect on patient health when they are exposed to overdiagnosis or overtreatment. Overdiagnosis occurs "when a disease is diagnosed in a person when it will never be symptomatic or fatal" [6] and could lead to a subsequent risk of overtreatment, exposing healthy patients to adverse effects of treatments with little or no benefit [7]. Berwick et al*.* defined overtreatment as “the waste that comes from subjecting patients to care that, according to sound science and the patients’ own preferences, cannot possibly help them” [8]. This issue is also financial, resulting in the unnecessary overuse of resources. Medical overuse accounted for 30% of healthcare costs in the United States in 2009 [9] and overtreatment was estimated to represent at least 6% of healthcare spending in 2011 [8]. This has also been observed in France: the French population was one of the world's largest consumers of drugs, notably the third largest consumer of antibiotics, in Europe in 2018 [10].

Multiple ways to address medical overuse have been developed. The Choosing Wisely campaign, launched by the American Board of Internal Medicine (ABIM) Foundation in 2012, aims to encourage dialogue between doctors and their patients on avoidable acts to reduce their prescription [11]. The principle is based on the creation of lists of five commonly prescribed care procedures (tests, treatments, interventions) that are not supported by evidence and potentially harmful to patients [11]. These lists, called top five lists, have been established in approximately 20 countries for more than 80 specialities [12]. Each list is specific to a specialty and a country. Seven countries have composed such lists in general practice: the United States [13], Canada [14], Switzerland [15], Australia [16], Italy [17], the United Kingdom [18], and France [19]. These lists are all different. None involved the input of patients, despite the recommendations published by teams of methodologists, who advocate soliciting patients for the determination of top five lists [20]. Health is often in the news in France. The French are very interested in this issue and the results of this study reflect this.

The main objective of this study was to select the first general practice top five list established by patients in France. The secondary objective was to understand the reasons for the patient choices.

## Method

**Fig. 1:**
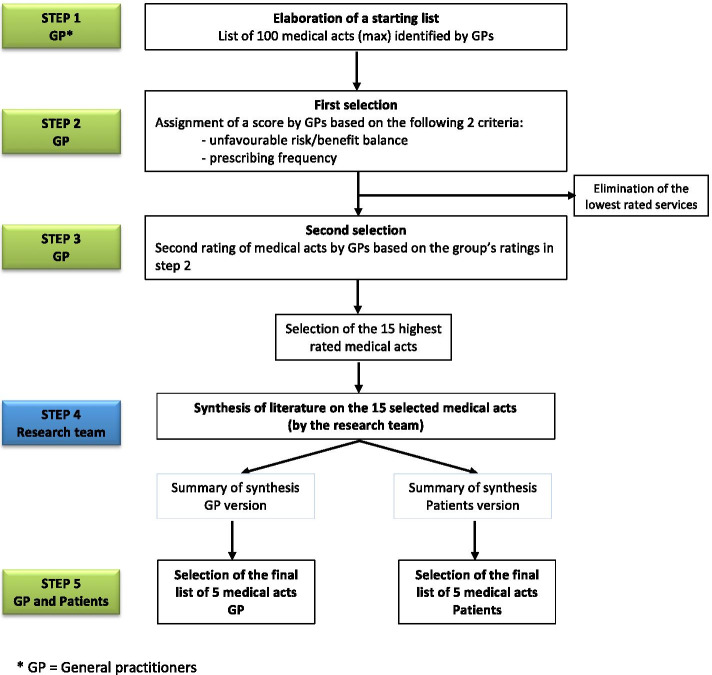
Schema of study, French top five list in general practice.* GP = General practitioners

This study is part of a larger project that also included determination of the French top five list in general practice by GPs [19]. The design of the project is presented in Fig. 1 and the protocol has been previously published [21]. The first four steps were common in the establishment of both top five lists (GPs and patients). In the first step, 40 GPs selected 100 care procedures considered to be involved in medical overuse. Steps two and three consisted of two Delphi rounds among the GPs, which permitted selection of the 15 care procedures considered to be the most relevant to medical overuse. The fourth step consisted of a literature review for each of the 15 care procedures to write 15 synthetic "guides". Some care procedures were associated with several indications. Literature reviews were conducted for each indication. These guides were first written for GPs and contained didactic information about the efficacy and safety of the care procedure. They also contained the current French recommendations for the care procedure, French or international data on the prevalence and incidence of the condition, and the cost to the French national health service [19]. These guides were concise, as homogenous as possible, and impartial in terms of the presented data. They were proofread by eight academic GPs for clarity and homogeneity. Three of the guides have been published [22–24]. To implement construction of the patients’ top five list, two independent researchers adapted the 15 guides to meet requirements related to patients' level of health literacy. The resulting guides were proofread and corrected by expert patients belonging to the "Pemphigus pemphigoïde France" association, which works on written and production of materials for patients with low health literacy language. The finalized guides were reviewed and validated by the study scientific council, which included study investigators, epidemiologists, methodologists and computer scientists. An example of the content of the "patient" guides is available in Additional file 1: Appendix 1.

### Study population

The patient panel was drawn from the web-based GrippeNet.fr cohort, which was launched during the 2011/2012 influenza season to study influenza-like illnesses in the French general population each winter [25]. This cohort occasionally participates in studies on subjects other than influenza [26]. For each participant, data, such as socio-demographic and health characteristics, are available through a background questionnaire that GrippeNet.fr participants complete each year.

A call for participation was sent by email to the GrippeNet.fr participants over 18 years of age who completed a profile survey for the 2018/2019-winter season. Questionnaires from participants who had consulted a GP at least once in the previous 12 months were retained to collect opinions from patients followed in general practice. Study participants could not be identified from the data collected in the study.

### Process of the vote

The 15 care procedures, with their guides, were presented to the participants in a random order on a secured web page developed for the study. Participants were asked to select the five most relevant care procedures in terms of medical overuse and for which the number of prescriptions should be reduced as a priority in general practice. To select a care procedure, patients had to open the corresponding guide (when they clicked on a procedure, the corresponding guide was displayed). Once the five care procedures were chosen, participants had to rank them from one (most unfavourable) to five (least unfavourable).

Once the top five list has been chosen, a short questionnaire was proposed to the participants. Questions focused on the participants' use of the proposed care procedures and various criteria specified in the guides that could have influenced their choices: health authority recommendations, effectiveness, tolerance, and the cost to health insurance. Data were collected using Likert-scale questions. The questionnaire is presented in Additional file 1: Appendix 2.

### Statistical analyses

The final patient top five list points to a care procedure depending on its ranking: the first choice received 5 points, the second one 4 points, and so on. The points for each care procedure were then added and the five items with the highest score constituted the top five list.

The distribution of variables related to the criteria that influenced the participants’ choices is presented using numbers and proportions.

Three sensitivity analyses were carried out to select the final top five list: the first according to the number of times the care procedures were quoted, regardless of the rank chosen by the participants; the second with sub-group analyses by gender (male *vs* female), age (less than 60-years *vs* 60-years-old or more), and educational level (high school or less *vs* more than high school) of the participants; and the third according to the number of guides consulted by the participants to observe whether reading a guide influenced their choice.

All statistical analyses were performed using R version 1.2.5001.

## Results

### Population characteristics

Between July 11 and September 1^st^, 2019, 6,449 individuals from the GrippeNet.fr cohort received an email to participate in the current study. Among them, 889 chose a top five list (participation rate: 14%). Participants who did not complete the short questionnaire after choosing their top five list (*n* = 71), those who did not complete the GrippeNet.fr annual background questionnaire (*n* = 1), and those who did not see a GP in the previous 12 months (*n* = 126) were excluded from the analyses. The final study population included 691 participants. Their characteristics are presented in Table 1. Among the participants, 55% were over the age of 60 and 65% were women. Concerning their health, 31% were undergoing at least one treatment for a chronic condition.

**Table 1:** Socio-demographic and health characteristics of GrippeNet.fr participants (*N* = 691)

	n	%
**Age (in years) (m.d **^**a**^**. = 1)**		
18–29	11	1.6
30–39	60	8.7
40–49	93	13.5
50–59	144	20.9
60–69	199	28.9
70 and over	181	26.3
**Gender**		
Female	450	65.1
Male	241	34.9
**Level of education (m.d. = 5)**	**m.d. = 5**	
No formal qualification, GCSE’s levels, CSEs or equivalent	111	16.2
A-levels or equivalent	116	16.9
Bachelor’s degree or equivalent	227	33.1
Higher Degree or equivalent	232	33.8
**Main activity**		
Working	311	45.0
Stay at home, looking for work or on sick or parental leave	33	4.8
Retired	332	48.0
Student, or other situation	15	2.2
**Treatment for a chronic disease**		
No	475	68.7
Yes	216	31.3
*Asthma*	*52*	*24.1*
*Diabetes*	*39*	*18.1*
*Pulmonary diseases*	*31*	*14.4*
*Heart diseases*	*102*	*47.2*
*Kidney diseases*	*5*	*2.3*
*Immunodeficiency*	*38*	*17.6*
**Tobacco use**		
No	628	90.9
Yes, occasionally or < 10 cigarettes/day	38	5.5
Yes, ≥ 10 cigarettes/day	25	3.6
**Body Mass Index (BMI Kg/m**^***1***^**) (m.d. = 2)**		
Underweight	19	2.7
Normal weight	357	51.7
Overweight	215	31.1
Obese	98	14.2

^a^ m.d. missing data

### Patients’ top five list

The top five list in general practice, established by patients consisted of:

1. Prescription of antibiotics for acute bronchitis, nasopharyngitis, otitis media with effusion, or uncomplicated influenza.
2. Prescription of benzodiazepines and benzodiazepine-like agents for insomnia, generalised anxiety, and all indications for older patients.
3. Prescription of a homeopathic treatment (Influenzinum) for flu prevention.
4. Prescription of antitussive or expectorant agents for acute cough or acute bronchitis care.
5. Prescription of statins for the primary prevention of cardio-vascular risk for older patients.

The ranking of care procedures did not change depending on the method used (the most cited item or the sum of the points awarded to the procedure (Table 2).

**Table 2:** Details and ranking of the 15 care procedures used to define the patients’ top five list

Care procedures	Indication	Patients’ top five list”			
		**Sum of points**		**Number of citations**	
		**Rank**	**Result**	**Rank**	**Result**
**Prescription of antibiotics**	*Acute bronchitis, nasopharyngitis, otitis media with effusion, uncomplicated influenza*	**1**	1,539	**1**	431
**Prescription of benzodiazepine and benzodiazepine-like agents**	*Insomnia, generalised anxiety and in older patients for all indications*	**2**	1,242	**2**	389
**Prescription of an homeopathic treatment (Influenzinum)**	*Flu prevention*	**3**	1,230	**3**	351
**Prescription of antitussive or expectorants agents**	*Acute cough or acute bronchitis care*	**4**	1,000	**4**	313
**Prescription of statins**	*Primary prevention of cardio-vascular risk in older patients*	**5**	960	**5**	301
Prescription of Tramadol or tramadol with paracetamol	*Pain care in older patients*	**6**	764	**6**	266
Prescription of lumbar scanner	*Low back pain evolving for less than 6 weeks*	**7**	684	**7**	259
Prescription of a non-steroidal anti-inflammatory agent (NSAID)	*Symptomatic treatment of acute sinusitis and pharyngitis*	**8**	670	**8**	254
Long term prescription of proton pump inhibitors	*Without reviewing the indication*	**9**	657	**9**	246
PSA Testing	*Systematic screening of prostate cancer in men older than 50 with no information given to the patient regarding the benefits and risks*	**10**	411	**10**	153
Mammography	*Systematic screening for breast cancer in women with no information given to the patient regarding the benefits and risks*	**11**	337	**11**	119
Prescription of cholinesterase inhibitors and memantin	*Mild cognitive impairment and Alzheimer disease*	**12**	289	**12**	113
Prescription of vasodilatator agents	*Peripheral Arterial Disease*	**13**	237	**13**	100
Prescription of DPP4 inhibitors	*Type 2 diabetes*	**14**	195	**14**	84
Prescription of allopurinol	*Asymptomatic hyperuricemia in prevention of gout attack, renal hypertension, cardio-vascular disease*	**15**	180	**15**	76

Sub-group results are presented in Tables 3 and 4. In the sub-group analyses of the number of guides consulted, the top five list differed from a single care procedure depending on whether the participants consulted all the guides or less than 10.

**Table 3:** Patients’ top five list among 15 care procedures, with sub-groups analyses by gender, age, and level of education

	**TOTAL** **(*n* = 691)**	**Gender**		**Age, in years**		**Level of education**	
		**Male** **(*n* = 241)**	**Female** **(*n* = 450)**	**Under 60** **(*n* = 308)**	**60 and over** **(*n* = 380)**	**High school or less (*n* = 227)**	**College or more (*n* = 459)**
**Antibiotics**	**1**	**1**	**1**	**1**	**1**	**1**	**1**
**Benzodiazepines**	**2**	**3**	**2**	**2**	**3**	**3**	**2**
**Homeopathic**	**3**	**2**	**3**	**3**	**2**	**2**	**3**
**Antitussive or expectorant**	**4**	**5**	**4**	**4**	**5**	**4**	**4**
**Statins**	**5**	**4**	**5**	**5**	**4**	**5**	**5**
Tramadol	6	9	6	9	5	6	6
NSAID^a^	7	8	9	6	8	8	9
Lumbar scanner	8	7	7	7	6	7	8
Proton pump inhibitors	9	10	8	8	7	9	7
PSA^b^	10	6	12	10	9	10	10
Mammography	11	11–12 ex aequo	10	11	10	11	11
Cholinesterase inhibitors	12	11–12 ex aequo	11	12	11	12	12
Vasodilatator agents	13	13	13	13	12	13	13
DPP4 inhibitors^c^	14	14	15	14	14	15	14
Allopurinol	15	15	14	15	13	14	15

^a^ Non-steroidal anti-inflammatory

^b^ Prostate specific antigen

^c^ Dipeptidyl peptidase 4 inhibitors

**Table 4:** Patients’ top five list” among 15 care procedures with sub-groups analyses according to the number of consulted guides

Care procedures	TOTAL (*n* = 691)	Among the participants who opened all the guides (*n* = 106)	Among the participants who opened only 5 guides (*n* = 271)	Among participants who opened at least 10 guides (*n* = 183)
**Antibiotics**	**1**	**1**	**1**	**1**
**Benzodiazepine**	**2**	**4**	**3**	**3**
**Homeopathic**	**3**	**2**	**2**	**2**
**Antitussive or expectorant**	**4**	8	**4**	**5**
**Statins**	**5**	**3**	**5**	**4**
Tramadol	6	7	6	7
Lumbar scanner	7	**5**	8	6
NSAID^a^	8	9	7	8
Proton pump inhibitors	9	6	9	9
PSA^b^	10	10	11	10
Mammography	11	14–15 ex aequo	10	14
Cholinesterase inhibitors and memantin	12	11	12	12–13 ex aequo
Vasodilatator agents	13	13	13	12–13 ex aequo
DPP4 inhibitor^c^	14	12	14	11
Allopurinol	15	14–15 ex aequo	15	15

^a^ Non-steroidal anti-inflammatory

^b^ Prostate specific antigen

^c^ Dipeptidyl peptidase 4 inhibitors

## Reasons for choosing items on the top five list

The distribution of the criteria that influenced the participants’ choices is presented in Table 5. More than 70% of participants responded that they gave importance to the recommendations of the health authorities, effectiveness, and tolerance to the medical care procedures. Almost half of respondents reported that the cost of the care procedure was not a factor in determining their choice.

**Table 5:** Distribution (n, %) of factors that influenced the choice of the top five list” from the GrippeNet.fr participants (*N* = 691)

	**n**	**%**
*Factors influencing the choice of FIVE*		
**Recommendations of the health authorities (HAS, WHO …) (m.d.**^**a**^** = 25)**		
Not at all	79	11.9
Rather not	109	16.4
Rather yes	336	50.4
Absolutely	142	21.3
**Effectiveness of the medical care (m.d = 30)**		
Not at all	43	6.5
Rather not	67	10.1
Rather yes	333	50.4
Absolutely	218	33.0
**Tolerance to the medical care (m.d. = 25)**		
Not at all	53	8.0
Rather not	128	19.2
Rather yes	306	45.9
Absolutely	179	26.9
**Cost of the care procedure (m.d. = 28)**		
Not at all	140	21.1
Rather not	182	27.4
Rather yes	216	32.6
Absolutely	125	18.9
**Personal history with study care procedure(s) (m.d. = 21)**		
No, never	112	16.2
Yes, for himself only	352	48.1
Yes, for a close relative only	68	9.8
Yes, for himself and for a close relative	158	22.9
**If yes, carrying out examinations or taking treatments (m.d. = 4)**		
No	12	2.2
Not every time	212	38.0
Yes every time	261	46.8
**If yes, have you included these care procedures in your “Top 5”? (m.d. = 18)**		
Yes, 1 only	185	33.2
Yes, 2	173	31.0
Yes, 3	72	12.9
Yes, 4	21	3.8
Yes, the 5	28	5.0
No, none	63	11.3

^a^ m.d. = missing data

Approximately 83% of the participants were prescribed, personally or for a relative, at least one of the care procedures presented in this study. Among them, 47% reported having taken the treatment or having undergone the medical exam for each prescription (e.g. Table 5).

## Discussion

This study led to the establishment of the first patients’ top five list. Only five American and Canadian published top five lists have involved patients in their development, despite the recommendations for developing these types of lists among lay individuals [20], but none in general practice. However, patient participation was limited to comments on the final list already established by American or Canadian rheumatologists, neurologists, and gastroenterologists. They did not participate in the final vote [27–30]. For American neonatal medicine, family representatives participated in the creation of the first list to identify medical overuse care procedures [31].

Consultation of the literature guides did not appear to be the only determinant in the selection of the top five list items. This may reflect pre-study opinions that cannot be changed by reading scientific material. More than 70% of the study patients had been exposed to at least one of the proposed care procedures. This is consistent with a French poll that reported that 77% of French individuals have a first opinion on their experience and background [32]. However, it is difficult to estimate the share of the a priori vision that the patients may have had for each care procedure. Finally, we did not seek to recruit patients specifically affected by an indication for which the procedure of care was in question. The purpose of this study was to get away from personal experience by reading the guidelines that provide scientific evidence.

Various information campaigns and controversies recently raised in France may have influenced the top five list as well. First, since 2002, the French population has been exposed to various health insurance campaigns aiming to reduce antibiotic consumption. After a 26.5% decrease in antibiotic prescriptions in France between 2002 and 2007 [33], an increase was observed in 2009. This led to a new series of awareness-raising campaigns on antibiotic resistance, which have stabilised antibiotic consumption, although France still used 35% more antibiotics than the European average in 2019 [34]. Concerning benzodiazepines, a French study published in 2014 was widely covered by the media [35]. It showed a positive association between the use of benzodiazepines and the development of dementia. Although these data were called into question in another study in 2015 [36], distrust of benzodiazepines and fear of developing Alzheimer's disease may have remained in the collective memory. Nevertheless, France remains the second largest consumer of benzodiazepines in Europe [37]. Concerning homeopathy, the Haute Autorité de Santé (HAS) decided in June 2019 to remove homeopathic products from the list of medicines reimbursed by the national health insurance, leading to a public debate widely covered by the media [38]. This has also been the case for the use of statins for primary prevention. The "shock" report on the use of statins broadcast on television in December 2017 relaunched a debate already initiated in 2013 by a French doctor with his polemical book "The truth about cholesterol", in which he refuted the benefit of statins as a means of primary prevention [39]. It led to a 40% increase in the proportion of patients stopping statin use within nine months of the book's publication [40]. This “shock” report disturbed the scientific community of cardiologists, who accused the media of establishing irrational and anxiety-provoking conspiracy theories [41]. In 2006, the delisting of mucolytics and expectorants from the list of medicines reimbursed by the national health insurance led to a 50% reduction in the prescription of these medications by GPs [42]. Since 2009, a re-evaluation of the indications for cough-suppressant therapy has been ongoing in France. In 2010, mucofluidifiers and mucolytics and, in 2011, first-generation anti-histamines and fenspiride were contraindicated for children under two years of age [43]. In 2015, codeine-based cough suppressants were contraindicated for children under 12 years of age [44]. These various waves of delisting may have made patients aware of the dangers associated with certain medications and the lack of efficacy of others. Conversely, anticholinesterase inhibitors and memantine were delisted in 2018 and do not appear in this classification.

Three items were common to the top five list produced by French GPs [19] and that by patients: antibiotic prescriptions, benzodiazepine prescriptions, and statin prescriptions. The first two items are already targeted by the French healthcare system for the reduction of prescriptions because of the high level of prescription in France relative to that of other European countries. Reductions in antibiotic and benzodiazepine prescriptions have already been addressed in four [13–15, 17] and two [17, 18] top five lists in general practice, respectively.

Additionally, data from the complementary questionnaire showed that participants had confidence in the recommendations made by various health authorities, although, according to a study, French confidence in the health authorities appeared to have been shaken over the last several years. Indeed, 52% of 1,000 French individuals put their trust in the public authorities and 33% in the pharmaceutical industry in 2019 [45].

Finally, cost was considered by only half of the patients. The French public was already aware of the cost of the health system in 2011 in an IPSOS survey, both at the individual and collective level. The survey showed that French people considered "lack of civic-mindedness" as the main explanation for the increase in healthcare spending (abuse/fraud and unnecessary medical acts for 70 and 59% of the respondents, respectively) [46].

The originality of this study lies in the participation of patients: it is the only top five list created exclusively by patients. The GrippeNet.fr cohort represented several advantages for this project, such as the presence of socio-demographic information, a high interest of participants in public health issues, and the high motivation of participants to participate in new research projects [25]. Additionally, the complementary questionnaire provided information to highlight their choices. Another important strength of this study was that the guides were written with the help of a patient-expert association to ensure that all participants could understand them. The guides created for the purpose of this study could serve as a basis for the creation of adapted shared decision support tools that would be interesting to implement in daily practice. Other studies should be considered to evaluate the impact of such tools on changes in prescribing habits. The three common procedures for the physician and patient lists seem to be a good starting point for effective actions.

This study also had several limitations. First, inherent to volunteer-based cohorts, the GrippeNet.fr population is not representative of the general population. Participants were older, more frequently women, had a higher level of education, and were probably more interested in health issues than the general population [25]. The impact of this potential selection bias is difficult to estimate and is frequently reported [47], although the top five list created by sub-group (age, gender, and level of education) provides reassuring results. This sample, despite its limitations, has the strength for the purpose of the study of being composed of patients who may be in frequent contact with general practice since about one third of them have a chronic disease. Second, in this study, patients were questioned based on the 15 care procedures chosen by the GPs and did not participate in the first pre-selection, which obviously influences the final results. This approach was chosen because patients appear to be unfamiliar with the notion of medical overuse and the concept of over-diagnosis itself is generally misunderstood by patients [48, 49]. Asking them to provide examples would have been difficult. Third, for some care procedures, the different indications were grouped together in a single guide (e.g. antibiotics) but could lead to different answers in terms of benefit/risk balance.

## Conclusion

This study provides the first top five list in general practice established by patients. It is specific to French healthcare issues. Three care procedures are common between the top five lists of GPs and patients: antibiotic prescriptions, benzodiazepine prescriptions, and statin prescriptions. Efforts can now be focused on these three procedures to reduce their prescription in France. It will be easier to establish actions, such as the development of shared decision support tools, which would be a first step towards initiating dialogue between GPs and patients.

## Supplementary Information

**Additional file 1.**

## Data Availability

All the data of this work are accessible on simple request to the authors.

## Abbreviations

ABIM: American Board of Internal Medicine
CCTIRS: Comité Consultatif sur le Traitement de l’Information en Matière de Recherche
CNIL: Commission Nationale de l’Informatique et des Libertés
GP: General practitioner
HAS: Haute Autorité de Santé
