# Supplementary material for: Implementation of a top five list to identify medical overuse in general practice according to patients’ viewpoint in 2019 in France
Source: BMC Fam Pract. 2021 Jun 26;22:134. doi: 10.1186/s12875-021-01475-z (PMC8235619; doi:10.1186/s12875-021-01475-z)
Supplement: Supplementary file 1 — Additional file 1. [file 12875_2021_1475_MOESM1_ESM.docx]

**Implementation of a top five list to identify medical overuse in general practice according to patients’ viewpoint in 2019 in France**

Agnès HAZARD^1*^, Marion DEBIN^2^, Corentin HERVE^2^, Caroline GUERRISI^2^, Camille BONNET^2^ and Mathilde FRANÇOIS^1,3^

^1^Department of Family Medicine, Faculty of Health Sciences Simone Veil, University Versailles-Saint-Quentin-en-Yvelines, Villejuif, Paris, France

^2^Sorbonne Université, INSERM, Institut Pierre Louis d’Epidémiologie et de Santé Publique, IPLESP F-75012, Paris, France.

^3^Centre for Research in Epidemiology and Population Health, French National Institute of Health and Medical Research (INSERM U 1018), University Versailles Saint-Quentin-en-Yvelines, University Paris-Sud, Villejuif, France.

*Corresponding Author: Agnès Hazard, [agneshazard@hotmail.com](mailto:lucie.fournier@iplesp.upmc.fr), phone: +33689185609

N°ORCID : [0000-0002-5936-1738](javascript:popup_orcidDetail(%22https://orcid.org%22,%20%220%22);)

Appendix 1: example of patients’ guide: Antibiotics in Acute Bronchitis, Uncomplicated Influenza, Seromucous ear infections and Acute Rhinopharyngitis


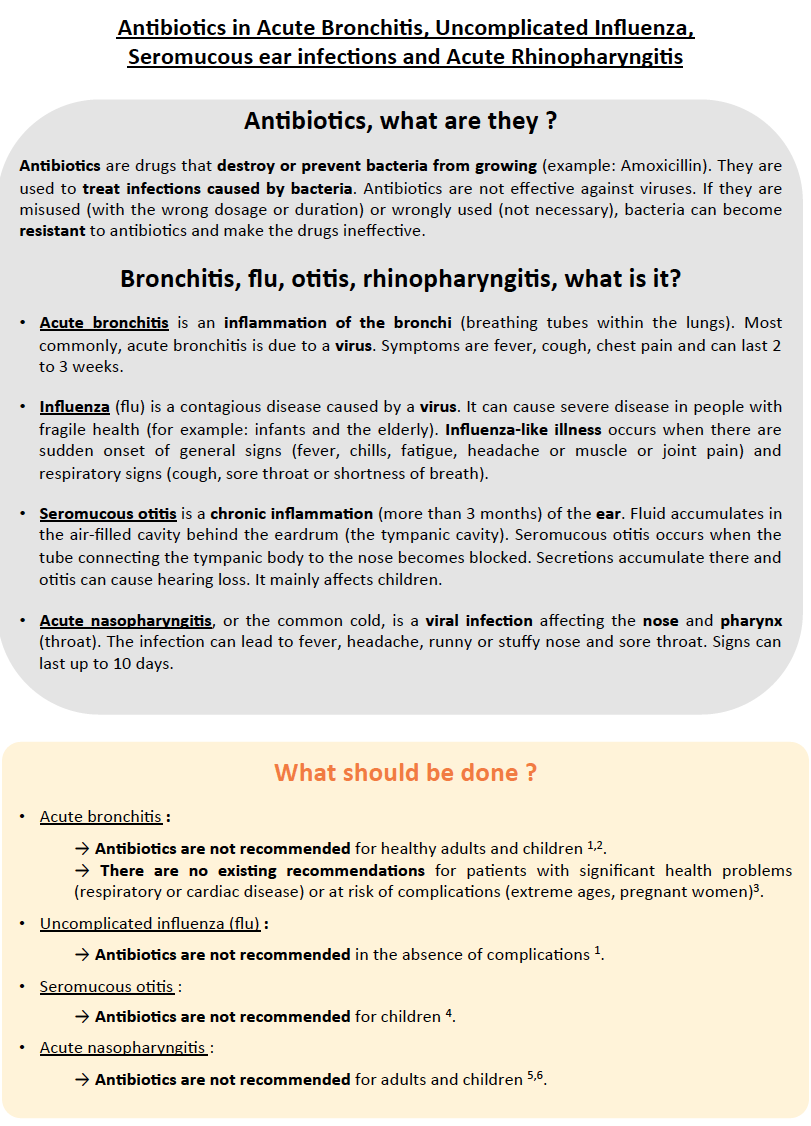


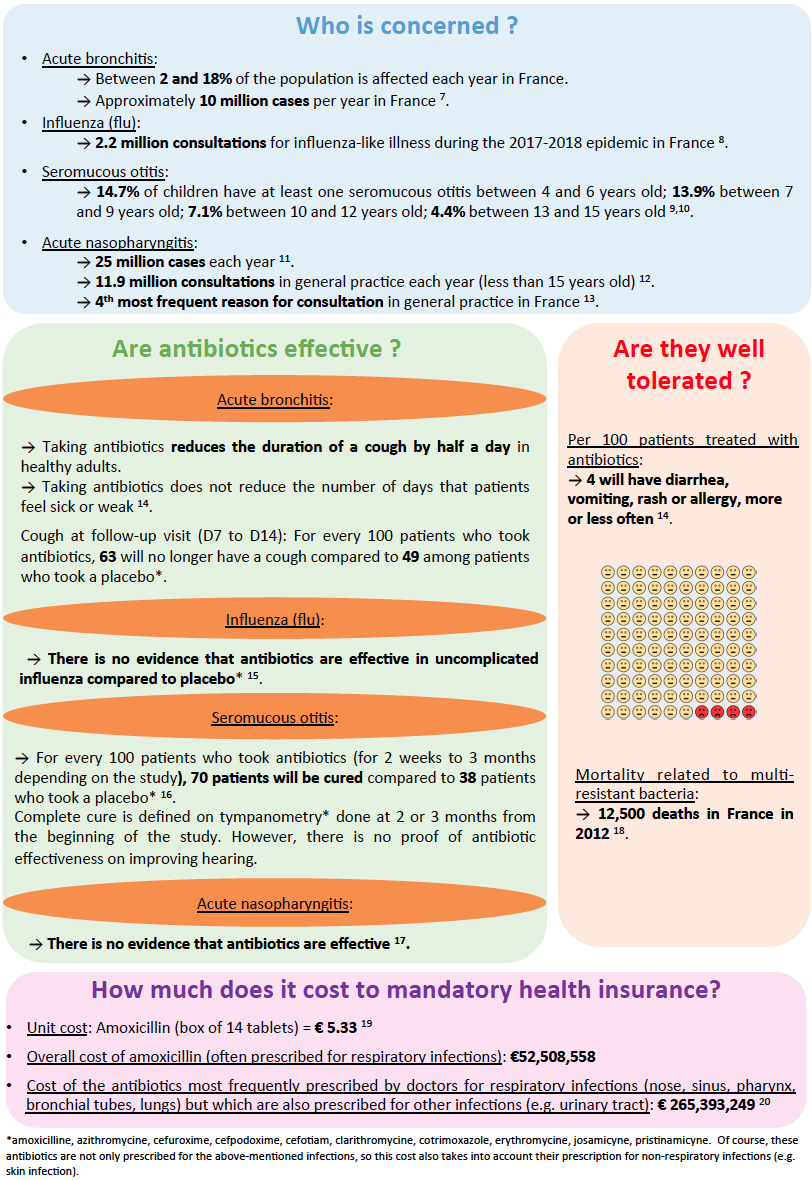


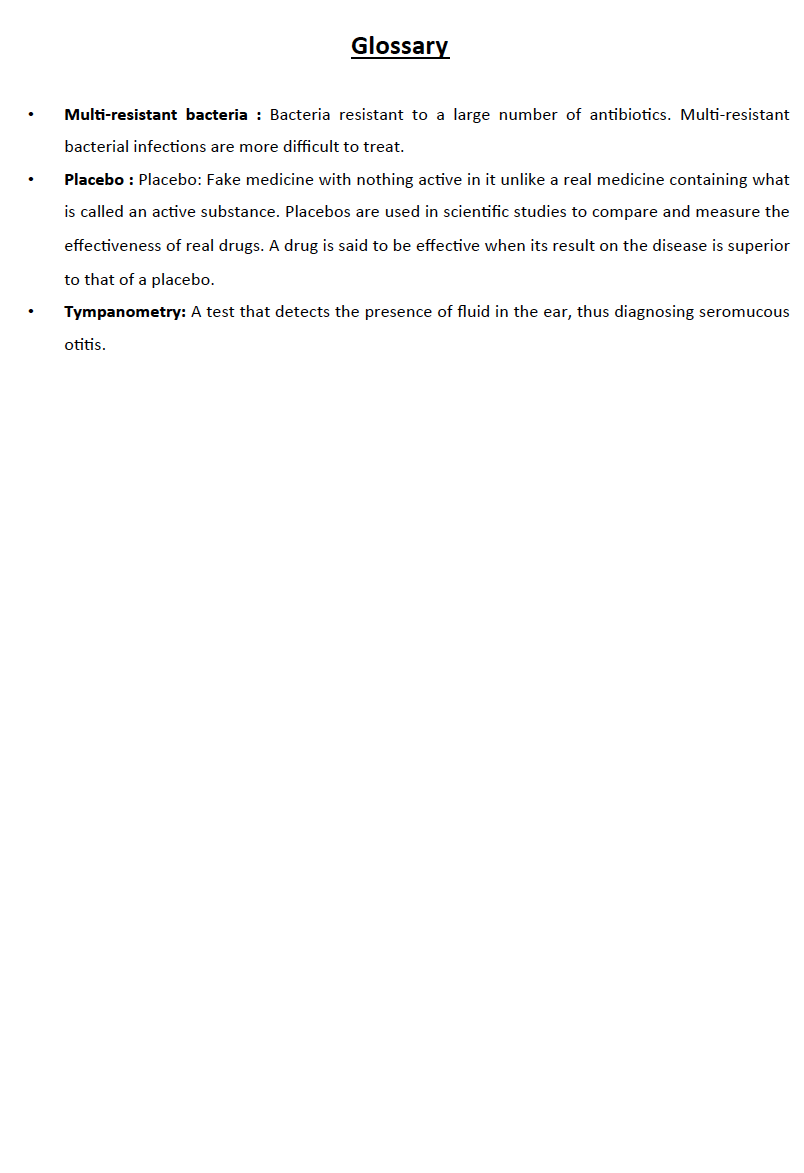


Appendix 2: questionnaire to ask patients about their choice of top five list.

| 1. Have you consulted a general practitioner for yourself in the last 12 months? (several possible answers) | | |
| --- | --- | --- |
|  | a. Yes, for you | |
|  | b. Yes, to help a relative | |
|  | c. Yes, to help someone who is not a relative | |
|  | d. No | |
|  | e. Don’t want to answer | |
| 2. Did you give importance to the following information to make your "Top 5"? | | |
|  | a. The recommendation of the various health authorities (HAS, WHO...) under the heading "what to do" | |
|  |  | i. Not at all |
|  |  | ii. Rather no |
|  |  | iii. Rather yes |
|  |  | iv. Absolutely |
|  |  | v. Don’t want to answer |
|  | b. The effectiveness of the care procedure (tests, treatments, examinations that benefit patients in terms of improving quality of life, reducing mortality and/or reducing the risk of disease) in the "Is it effective?" section | |
|  |  | i. Not at all |
|  |  | ii. Rather no |
|  |  | iii. Rather yes |
|  |  | iv. Absolutely |
|  |  | v. Don’t want to answer |
|  | c. The tolerance of the care procedure (tests, treatments, examinations causing adverse reactions or serious side effects) under the heading "Is it well tolerated?” | |
|  |  | i. Not at all |
|  |  | ii. Rather no |
|  |  | iii. Rather yes |
|  |  | iv. Absolutely |
|  |  | v. Don’t want to answer |
|  | d. The cost of the treatment procedure (tests, treatments, examinations reimbursed by the health insurance each year) under the heading "How much does it cost?" | |
|  |  | i. Not at all |
|  |  | ii. Rather no |
|  |  | iii. Rather yes |
|  |  | iv. Absolutely |
|  |  | v. Don’t want to answer |
| 3. Have you ever been confronted in your personal life with some of these tests, treatments or exams? | | |
|  | a. No, I have never had a prescription for any of these tests or treatments | |
|  | b. Yes, I already had a prescription for one of these tests or treatments | |
|  | c. Yes, one of my relatives already had a prescription for one of these tests or treatments | |
|  | d. Don’t want to answer | |
| 4. If so, did you perform these tests or take these treatments? | | |
|  | a. No | |
|  | b. Not every time | |
|  | c. Yes, every time | |
|  | d. Don’t want to answer | |
| 5. If so, did you include these tests or treatments in your "Top 5"? | | |
|  | a. Yes, only 1 | |
|  | b. Yes, 2 | |
|  | c. Yes, 3 | |
|  | d. Yes, 4 | |
|  | e. Yes, the 5 | |
|  | f. No, none of them | |
|  | g. Don’t want to answer | |
